# Anti-PM/Scl Antibody-Positive Dermatomyositis With Rapidly Progressive Interstitial Lung Disease in a 19-Year-Old Male: Clinical Implications of a Presumptive Diagnosis Based on Line Immunoassay Positivity

**DOI:** 10.7759/cureus.103151

**Published:** 2026-02-07

**Authors:** Nozomi Kawamata, Kei Toumura, Takayuki Kon, Hirofumi Amano

**Affiliations:** 1 Internal Medicine and Rheumatology, Juntendo University Nerima Hospital, Tokyo, JPN

**Keywords:** anti-pm/scl antibody, dermatomyositis, mycophenolate mofetil, rapidly progressive interstitial lung disease, young adult

## Abstract

Anti-PM/Scl antibodies are classically linked to systemic sclerosis-polymyositis overlap, but a dermatomyositis phenotype with interstitial lung disease has also been described. However, the clinical course and therapeutic approach for short-term progressive interstitial lung disease in anti-PM/Scl-positive dermatomyositis remain incompletely defined. A 19-year-old Japanese male developed cough and exertional dyspnea after several weeks of progressive myalgia and proximal muscle weakness. Marked creatine kinase elevation, a high-titer nucleolar-pattern antinuclear antibody, Gottron papules, and mechanic’s hands were noted, and dermatomyositis was diagnosed based on muscle magnetic resonance imaging and skin histopathology. High-resolution chest computed tomography showed bilateral, lower-lobe-predominant ground-glass opacities with infiltrative changes, consistent with a subacute-to-rapidly progressive course of interstitial lung disease. Anti-PM/Scl-75 and PM/Scl-100 antibodies were positive by a line immunoassay. As immunoprecipitation was unavailable, orthogonal testing on a different platform (wet protein array) also demonstrated strong positivity, increasing diagnostic confidence. After methylprednisolone pulse therapy, followed by high-dose glucocorticoids, interstitial lung disease findings persisted, prompting initiation and up-titration of mycophenolate mofetil. Imaging abnormalities and serum Krebs von den Lungen-6 improved over time after mycophenolate mofetil initiation, although the independent effect could not be determined because multiple interventions overlapped. Intravenous immunoglobulin was added for residual myositis activity, leading to improvement in muscle enzymes and discharge. This case highlights that anti-PM/Scl-positive dermatomyositis in young adults can present with short-term progressive interstitial lung disease and illustrates a pragmatic diagnostic and therapeutic strategy when immunoprecipitation is not feasible.

## Introduction

Anti-PM/Scl antibodies are autoantibodies directed against the exosome complex (PM/Scl-75 and PM/Scl-100) and have classically been associated with overlap between systemic sclerosis and polymyositis [1]. In anti-PM/Scl-positive idiopathic inflammatory myopathy (IIM), interstitial lung disease (ILD) is frequently accompanied by myositis and arthritis, and a substantial number of patients present with dermatomyositis (DM)-like skin manifestations and are classified as DM [2]. However, the clinical course and therapeutic strategies for young adult-onset cases and for ILD that progresses over a short period in anti-PM/Scl-positive DM have not been sufficiently characterized [2].

Anti-PM/Scl antibodies are measured using assays such as line immunoassay (LIA); however, results may be discordant depending on the immunoassay used. Therefore, antibody results should be interpreted cautiously in conjunction with the clinical phenotype and other test findings. When necessary, re-evaluation using an alternative method should be considered.

Rapidly progressive interstitial lung disease (RP-ILD) refers to an ILD phenotype characterized by clinically significant deterioration in respiratory status and/or radiographic progression over a short period of time (typically weeks to a few months), often necessitating prompt escalation of immunosuppressive therapy. For progressive ILD associated with myositis, a treatment strategy combining high-dose glucocorticoids with an immunosuppressive agent has been proposed [3]. In addition, monitoring with pulmonary function testing and high-resolution CT (HRCT) is recommended to assess treatment response and to detect relapse [4].

Here, we report a case of anti-PM/Scl-positive DM in a 19-year-old man in whom anti-PM/Scl antibodies detected by LIA were orthogonally confirmed using a different platform and who developed ILD progressing over a short period as the major organ involvement. Because ILD findings persisted despite high-dose glucocorticoid therapy, mycophenolate mofetil (MMF) was introduced and up-titrated, after which ILD showed a trend toward improvement. We also describe the course in which intravenous immunoglobulin (IVIG) was added for smoldering myositis activity with subsequent improvement, and we discuss the significance of treatment selection and diagnostic strategy.

## Case presentation

A 19-year-old Japanese male with no notable past medical or family history presented for evaluation. In April of the year 2025, he developed myalgia in both lower legs, followed by a gradual progression of proximal muscle weakness in the extremities. Over the subsequent several weeks, a dry cough and exertional dyspnea developed, and he was referred to our hospital for further evaluation and treatment later that month.

**Figure 1:**
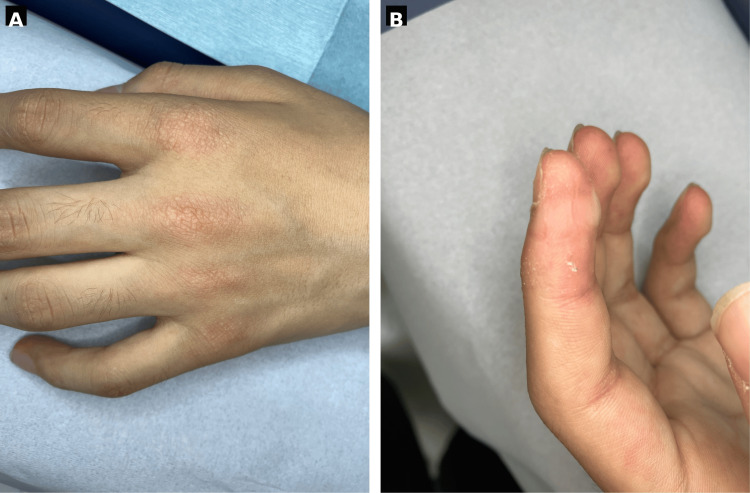
Cutaneous findings. (A) Gottron papules on the dorsal hands. (B) Mechanic’s hands on the fingers.

**Figure 2:**
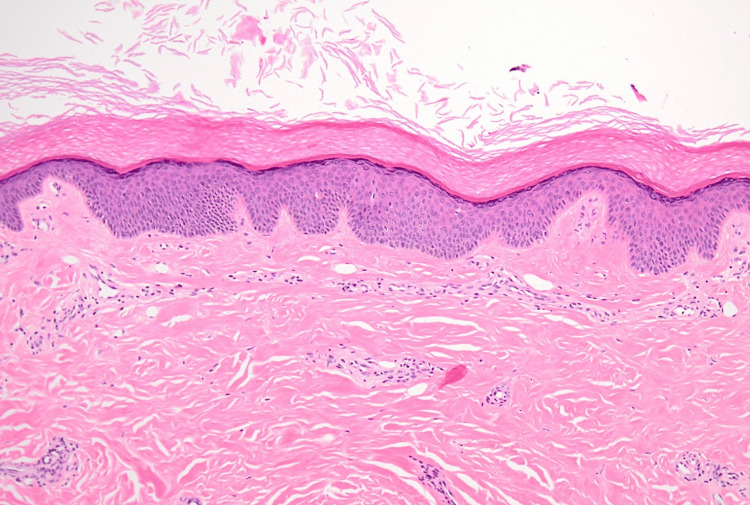
Skin biopsy of the dorsal hand lesion (hospital day five). Hematoxylin and eosin stain, original magnification ×100. The biopsy specimen was obtained from a Gottron papule. The epidermis shows mild acanthosis with hyperkeratosis. A sparse superficial perivascular lymphocytic infiltrate is present in the dermis. No dermal sclerosis, thickened/hyalinized collagen bundles, or adnexal entrapment suggestive of scleroderma is identified. The mild inflammatory infiltrate may have been attenuated by prior corticosteroid therapy.

On presentation, his height was 170 cm, weight was 50 kg, temperature was 36.7°C, blood pressure was 110/70 mmHg, pulse was 78 beats/min, and oxygen saturation (SpO₂) was 97% on room air. On physical examination, he had myalgia and proximal-predominant muscle weakness. Manual muscle testing (MMT) showed 3-4/5 strength in the proximal muscles of both the upper and lower limbs. Deep tendon reflexes were normal, and there were no sensory disturbances. Skin examination revealed Gottron papules on the dorsal hands (Figure 1A) and mechanic’s hands on the fingers (Figure 1B). In contrast, heliotrope rash and the V-neck sign were not evident. Skin sclerosis and Raynaud phenomenon were absent. Nailfold and periungual erythema and scaling with punctate hemorrhagic changes were observed. A skin biopsy of the dorsal hand rash showed findings consistent with Gottron papules (Figure 2).

Laboratory testing demonstrated markedly elevated muscle enzymes: CK10 = 781 U/L (reference range = 59-248 U/L), lactate dehydrogenase (LDH) = 1,749 U/L (reference range = 124-222 U/L), aspartate aminotransferase (AST) = 568 U/L (reference range = ≤30 U/L), and alanine aminotransferase (ALT) = 407 U/L (reference range = ≤30 U/L). CRP was mildly elevated at 1.21 mg/dL (reference range = ≤0.3 mg/dL). Ferritin was 508 ng/mL (reference range = 20-250 ng/mL), representing a moderate elevation that can be seen as a nonspecific inflammatory marker in inflammatory myopathies and myositis-associated interstitial lung disease. Krebs von den Lungen-6 (KL-6) was 952 U/mL (reference range = <500 U/mL). Thyroid function (FT3 = 3.2 pg/mL, FT4 = 1.3 ng/dL, thyroid-stimulating hormone = 1.5 μIU/mL) was normal. Antinuclear antibody was positive at a titer of 1:1280 with a nucleolar pattern.

Autoantibodies measured in routine clinical practice, including anti-ARS, anti-Mi-2, anti-MDA5, anti-TIF1-γ, anti-mitochondrial M2, anti-SSA/Ro, anti-U1 RNP, anti-centromere, anti-RNA polymerase III, and anti-topoisomerase I (Scl-70), were all negative. An LIA showed anti-PM/Scl-75 antibody at 3+ and anti-PM/Scl-100 antibody at 1+, while other myositis- and systemic sclerosis-related antibodies were negative. As false-positive results can be an issue with LIA, we performed orthogonal confirmation using a different platform, A-Cube® (multiplex wet protein array), to increase diagnostic confidence; this showed strong positivity with a PM/Scl-75 index of 254.0 and a PM/Scl-100 index of 170.2 (cutoffs: <7.0 negative, 7.0-10.0 borderline, ≥10.0 positive). Autoantibody results and assay methods are summarized in Table 1.

**Table 1:** Autoantibody results and assay methods. ANA titer is shown with the immunofluorescence staining pattern. Enzyme-linked immunosorbent assay (ELISA) and double immunodiffusion (DID; Ouchterlony method) results were interpreted as negative or positive according to the performing laboratory’s cutoffs. Line immunoassay (LIA) classes were interpreted as follows: class 0 = negative, class (+) = borderline, and class + to +++ = positive. A-Cube® (multiplex wet protein array assay) results are reported as index values with assay-specific cutoffs: standard antigens were interpreted as negative (<7.0), borderline (7.0–10.0), and positive (≥10.0), whereas SRP-related antigens were interpreted as negative (<10.0), borderline (10.0–25.0), and positive (>25.0). PM/Scl-75 and PM/Scl-100 are shown for both LIA and A-Cube® to demonstrate concordant positivity across different assay platforms.

Antibody	Result	Interpretation criteria	Assay/method
Antinuclear antibody (ANA)	1:1280	Positive (titer)	Nucleolar pattern
Anti-MDA5	Negative	Negative/Positive	ELISA
Anti-TIF1-γ	Negative	Negative/Positive	ELISA
Anti-Mi-2	Negative	Negative/Positive	ELISA
Anti-ARS	Negative	Negative/Positive	ELISA
Anti-mitochondrial M2 antibody	Negative	Negative/Positive	ELISA
Anti-SSA/Ro	Negative	Negative/Positive	DID
Anti-U1 RNP	Negative	Negative/Positive	DID
Anti-Scl-70 (topoisomerase I)	Negative	Negative/Positive	DID
Anti-centromere	Negative	Negative/Positive	ELISA
Anti-RNA polymerase III	Negative	Negative/Positive	ELISA
Anti-PM/Scl-75	3+	See legend	LIA
Anti-PM/Scl-100	1+	See legend	LIA
Anti-NOR90	Negative	See legend	LIA
Anti-Th/To	Negative	See legend	LIA
Anti-Ku	Negative	See legend	LIA
Anti-PDGFR	Negative	See legend	LIA
PM/Scl-75	Positive (Index 254.0)	Negative <7.0; Borderline 7.0-10.0; Positive ≥10.0	A-Cube®
PM/Scl-100	Positive (Index 170.2)	Negative <7.0; Borderline 7.0-10.0; Positive ≥10.0	A-Cube®
SRP54	Negative (Index 0.4)	Negative <10.0; Borderline 10.0-25.0; Positive >25.0	A-Cube®
SRP14	Negative (Index 0.3)	Negative <10.0; Borderline 10.0-25.0; Positive >25.0	A-Cube®
SRP19	Negative (Index 2.9)	Negative <10.0; Borderline 10.0-25.0; Positive >25.0	A-Cube®
SRP68	Negative (Index 2.4)	Negative <10.0; Borderline 10.0-25.0; Positive >25.0	A-Cube®
SRP72	Negative (Index 0.8)	Negative <10.0; Borderline 10.0-25.0; Positive >25.0	A-Cube®
SAE (SAE1)	Negative (Index 0.1)	Negative <7.0; Borderline 7.0-10.0; Positive ≥10.0	A-Cube®
SAE (UBA2)	Negative (Index 0.5)	Negative <7.0; Borderline 7.0-10.0; Positive ≥10.0	A-Cube®
NXP-2 (MORC3)	Negative (Index 0.3)	Negative <7.0; Borderline 7.0-10.0; Positive ≥10.0	A-Cube®
U2-RNP (SNRPB2)	Negative (Index 4.9)	Negative <7.0; Borderline 7.0-10.0; Positive ≥10.0	A-Cube®
RuvBL1/2	Negative (Index 0.4)	Negative <7.0; Borderline 7.0-10.0; Positive ≥10.0	A-Cube®
Ki	Negative (Index 2.1)	Negative <7.0; Borderline 7.0-10.0; Positive ≥10.0	A-Cube®

**Figure 3:**
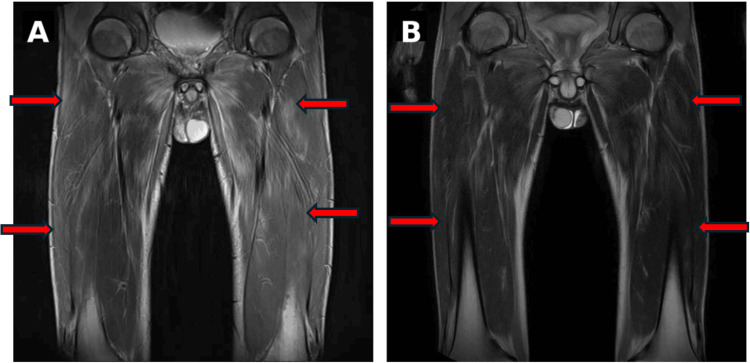
Thigh MRI. (A) Hospital day 1: Diffuse, symmetric T2 hyperintensity in the gluteal and thigh muscles (arrows). (B) Hospital day 28: Decreased T2 signal intensity in the corresponding muscles (arrows), consistent with improvement of myositis.

**Figure 4:**
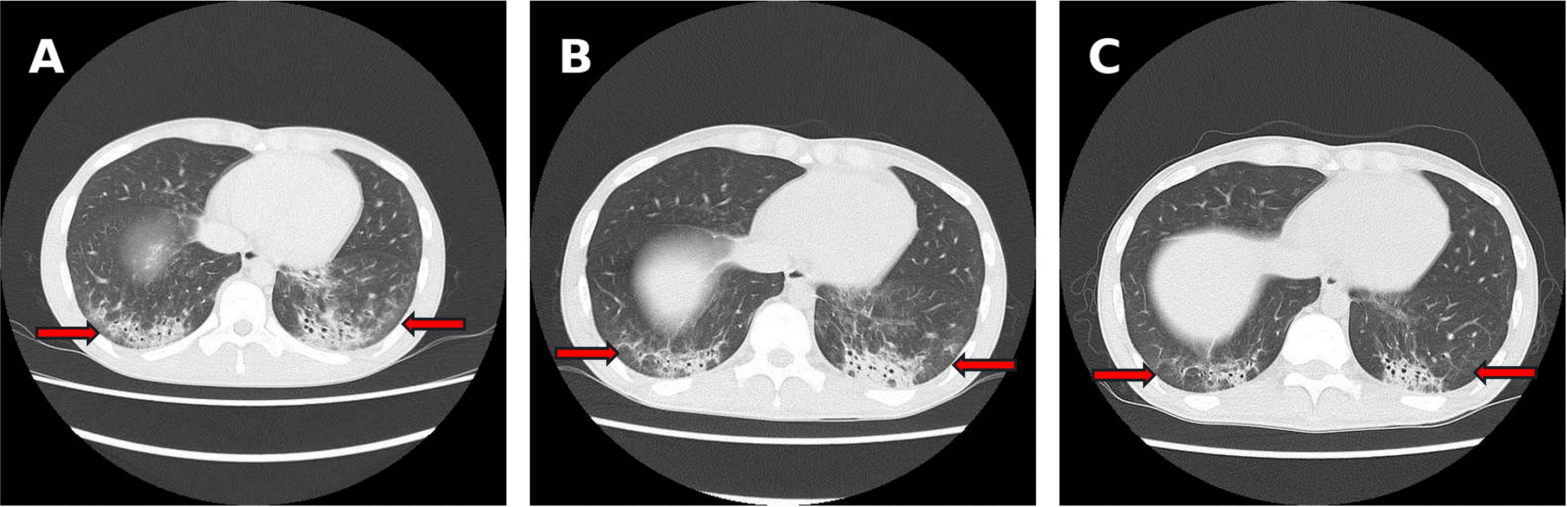
Chest CT (axial, lung window). (A) Hospital day 1: Bilateral, lower-lobe-predominant ground-glass opacities with patchy peribronchovascular/subpleural consolidation and mild reticulation (arrows), suggestive of an NSIP/OP overlap pattern. (B) Hospital day 13: Representative ground-glass opacities and patchy consolidations (arrows) persist after high-dose glucocorticoid therapy. (C) Hospital day 28: Decreased ground-glass opacities and consolidations (arrows) after initiation of mycophenolate mofetil. Abbreviations: NSIP, nonspecific interstitial pneumonia; OP, organizing pneumonia.

Imaging studies revealed extensive, bilaterally symmetric areas of T2 hyperintensity in the gluteal and thigh muscle groups on thigh MRI (Figure 3). High-resolution chest CT showed bilateral, lower-lobe-predominant interstitial abnormalities with ground-glass opacities and infiltrative shadows, suggesting a nonspecific interstitial pneumonia (NSIP)/organizing pneumonia (OP) overlap pattern (Figure 4). Pulmonary function testing demonstrated a restrictive ventilatory defect and reduced diffusing capacity (forced vital capacity (FVC) = 47.7%, diffusing capacity of the lungs for carbon monoxide (DLCO) = 42.0%). Cerebrospinal fluid examination was unremarkable, and electrophysiologic testing showed myopathic changes in the thigh muscles.

Based on these findings, the patient was diagnosed with dermatomyositis. Although strict time-series imaging comparisons were limited, ILD was clinically assessed as rapidly progressive or subacutely progressive because respiratory symptoms developed within several weeks, and extensive interstitial changes with pulmonary function impairment were present at the time of presentation.

**Figure 5:**
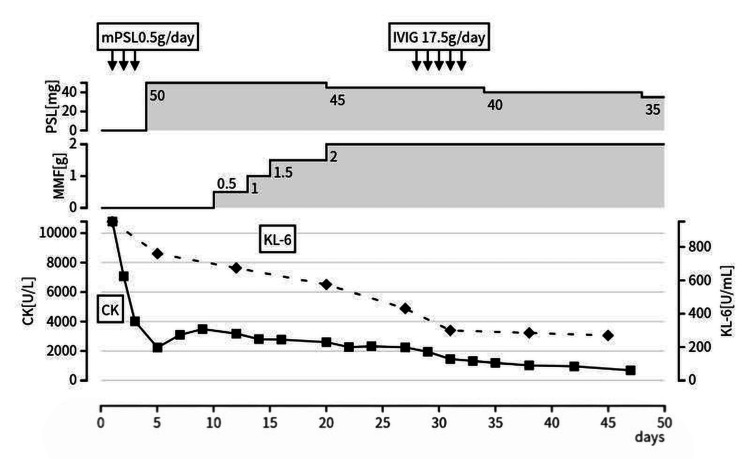
Clinical course. After mPSL 0.5 g/day for three days (arrows), PSL was tapered from 50 mg/day while MMF was initiated and up-titrated. ILD showed a trend toward improvement after MMF initiation; however, because smoldering CK elevation persisted, IVIG (17.5 g/day for five days) was added on hospital day 28. Serum CK is shown as solid squares (left y-axis), and KL-6 as dashed diamonds (right y-axis). The patient was discharged on hospital day 49. Abbreviations: ILD, interstitial lung disease; IVIG, intravenous immunoglobulin; CK, creatine kinase; KL-6, Krebs von den Lungen-6; MMF, mycophenolate mofetil; mPSL, methylprednisolone; PSL, prednisolone.

Methylprednisolone (mPSL) 500 mg/day was administered for three days starting on hospital day one, followed by prednisolone (PSL) 50 mg/day from hospital day four. Creatine kinase (CK) levels and imaging findings showed a trend toward improvement but were judged insufficient; therefore, MMF was added on hospital day 10 and gradually up-titrated. During the period after MMF initiation and dose escalation, both myositis and ILD findings improved (Figures 4, 5). However, because the decline in CK plateaued and residual myositis activity was suggested, IVIG 17.5 g/day for five days was added on hospital day 28. Thereafter, PSL was gradually tapered, and the patient was discharged on hospital day 49 (Figure 5). At approximately six months of outpatient follow-up after discharge, the patient has remained clinically stable without recurrence, and longer-term follow-up is ongoing.

## Discussion

Dermatomyositis phenotype in anti-PM/Scl-positive idiopathic inflammatory myopathy

Anti-PM/Scl antibodies were initially reported as autoantibodies characteristic of overlap syndromes between systemic sclerosis (SSc) and polymyositis; however, subsequent cohort studies have shown that, among patients with anti-PM/Scl-positive idiopathic inflammatory myopathies (IIM), a certain proportion present with DM-like rashes and meet classification criteria for DM [1,2]. Recent analyses have also reported that many anti-PM/Scl-positive IIM patients exhibit cutaneous manifestations, including DM-like rashes such as Gottron papules and heliotrope rash, suggesting that anti-PM/Scl-positive DM represents an important subgroup [1,2].

In the present case, the DM phenotype was evident based on typical Gottron papules and mechanic’s hands, proximal-predominant muscle weakness, inflammatory findings on muscle MRI, myopathic changes on electrophysiologic testing, and skin histopathology. Although periungual changes were observed, there were no SSc-typical features such as skin sclerosis, puffy fingers, or Raynaud phenomenon, and the presentation differed from a classic SSc overlap phenotype. Therefore, it is reasonable to position this case as “anti-PM/Scl-positive IIM with DM predominance” rather than “SSc overlap.”

Young adult onset and features of rapidly progressive ILD

Anti-PM/Scl-positive IIM is more common in adults, and cohort studies have reported a mean age of approximately 49 years, with onset typically in middle age [2]. Although young-onset cases have been described in case reports, most are small case series, and detailed clinical trajectories and long-term outcomes remain insufficiently characterized. ILD in anti-PM/Scl-positive disease is generally considered to be relatively treatment responsive and often slowly progressive; however, a subset of patients may experience progression over several weeks and be clinically assessed as having RP-ILD, as in the present case [5].

RP-ILD is classically observed in anti-MDA5-positive DM, for which intensive combination immunosuppressive therapy is often employed [6]. However, clear evidence has not accumulated on whether the same regimens should be uniformly applied to anti-MDA5-negative RP-ILD. This case exhibited an RP-ILD-like course despite being anti-MDA5 negative, suggesting that an aggressive pulmonary phenotype may exist within anti-PM/Scl-positive DM. The young adult onset in this case may be an important perspective for future case accumulation when examining the relationship between age and patterns of ILD progression.

Antibody testing strategy: LIA, A-Cube®, and the role of immunoprecipitation

Line immunoassays (LIA) are widely used to measure anti-PM/Scl antibodies. However, for some myositis-specific and myositis-associated autoantibodies, false positivity can be problematic; therefore, interpretation should be guided by consistency with the clinical phenotype and other autoantibody findings [1]. Recent international consensus statements recommend confirming at least high titers, or verifying results using immunoprecipitation or an alternative platform, for non-anti-ARS myositis-specific autoantibodies and myositis-associated autoantibodies [7,8].

In this case, the pretest probability of anti-PM/Scl positivity was considered high given a high-titer nucleolar-pattern antinuclear antibody, a DM phenotype, and concomitant ILD. After LIA demonstrated positivity for PM/Scl-75 and PM/Scl-100, we performed orthogonal confirmation using a multiplex wet protein array (A-Cube®) and confirmed strong positivity against both antigens. A-Cube was developed as a multiplex protein array targeting SSc- and PM/DM-related autoantibodies, and agreement with existing methods (including immunoprecipitation and enzyme-linked immunosorbent assay) has been reported, suggesting good specificity; however, large-scale validation specifically for anti-PM/Scl antibodies remains limited [9,10]. A major limitation of this case is the lack of confirmation by immunoprecipitation. Nevertheless, in a setting where the clinical phenotype and serologic background are strongly concordant, consistent anti-PM/Scl positivity across two different platforms (LIA and A-Cube) may serve as supportive evidence for the working diagnosis of anti-PM/Scl-positive DM. In routine practice where immunoprecipitation is not always available, a stepwise testing strategy-performing orthogonal confirmation on a different platform when LIA is positive in a high-risk phenotype-may be practical.

Treatment strategy for RP-ILD: MMF combination and rationale for avoiding cyclophosphamide

This case was considered DM-predominant anti-PM/Scl-positive IIM with RP-ILD as the major organ involvement; thus, treatment was discussed primarily in the context of myositis-associated ILD. In anti-MDA5-positive RP-ILD, intensive combination therapy with high-dose glucocorticoids (GC), calcineurin inhibitors, cyclophosphamide, and other agents is recommended [6]; however, a clearly established standard regimen for anti-MDA5-negative RP-ILD is lacking. The American College of Rheumatology (ACR)/American College of Chest Physicians (CHEST) guideline proposes a strategy for myositis-associated RP-ILD in which high-dose GC is administered after methylprednisolone pulse therapy, and one or two agents (depending on the clinical context) are selected from rituximab, cyclophosphamide, MMF, calcineurin inhibitors, Janus kinase (JAK) inhibitors, and IVIG [3]. In contrast, when anti-MDA5 positivity is suspected, intensive combination therapy adding calcineurin inhibitors and cyclophosphamide to high-dose GC is recommended [6,11].

In this case, anti-MDA5 antibodies were negative, there was no overt respiratory failure at admission, and the patient was young, making fertility preservation an important consideration. Cyclophosphamide has dose-dependent gonadotoxicity in both men and women; in particular, in young men, future infertility risks such as reduced sperm counts and azoospermia can be clinically significant [12,13]. Calcineurin inhibitors are reasonable steroid-sparing options for myositis-associated ILD, and we did not have a specific reason to avoid them in this case. However, given the anti-PM/Scl-associated overlap spectrum and the established use of MMF in systemic sclerosis-associated ILD [3], we selected MMF as the initial adjunct immunosuppressant, primarily based on (1) MMF being listed as a treatment option for myositis-associated RP-ILD in guidelines [3], (2) the desirability of avoiding cyclophosphamide from a fertility perspective [12,13], and (3) a low likelihood of anti-MDA5-associated disease (at least not a typical phenotype).

After MMF initiation, ILD showed a trend toward improvement, with improvement in imaging findings, KL-6, and pulmonary function. However, because MMF was administered in combination with high-dose GC, it is difficult to strictly isolate MMF’s independent contribution. Thus, the key message of this case is that, when RP-ILD is judged to respond insufficiently to high-dose GC alone, adding MMF can be a clinically rational decision based on individual patient background (fertility considerations) and serologic context (anti-MDA5 negativity).

IVIG has demonstrated efficacy as an add-on therapy for myositis [14,15]. In this case, after MMF initiation, the decline in CK plateaued, suggesting residual myositis activity; IVIG was then added, resulting in CK normalization and improved muscle strength. Although it is difficult to separate the contribution of each therapy in a multi-agent regimen, the response pattern, i.e., (1) insufficient response of both myositis and ILD to high-dose GC alone, (2) improvement of pulmonary involvement after MMF initiation with residual myositis activity, and (3) improvement of myositis after IVIG addition, supports an approach of tailoring additional therapy to organ-specific disease activity.

At approximately six months of outpatient follow-up after discharge, the patient has remained clinically stable without recurrence. Follow-up chest assessments were performed in routine care; however, additional chest images are not included in this report. When selecting immunosuppressive therapy for myositis-associated RP-ILD in young men, it is necessary to carefully balance survival benefit with the preservation of future reproductive function. This case represents an example in which favorable short-term outcomes were achieved by combining MMF and IVIG while considering cyclophosphamide-associated gonadotoxicity in RP-ILD complicated by anti-PM/Scl-positive DM. Further accumulation of similar cases is warranted to clarify pathophysiology and to refine treatment strategies.

## Conclusions

This case describes a young adult with anti-PM/Scl-positive dermatomyositis in whom ILD was the major organ involvement with a subacute-to-rapidly progressive course. Because pulmonary involvement persisted despite high-dose glucocorticoids, MMF was added, and IVIG was subsequently administered for residual myositis activity, leading to improvement in both myositis and ILD without relapse during follow-up. As multiple therapies overlapped, the independent contribution of each agent cannot be determined. When immunoprecipitation is not feasible, integrating the clinical phenotype with orthogonal confirmation using a different platform may improve diagnostic confidence. Treatment decisions for RP-ILD should incorporate guideline-listed options alongside individual factors such as age and future fertility.
